# Comments and Illustrations of the European Federation of Societies for Ultrasound in Medicine Guidelines: Benign Pleura Lesions (Benign Pleura Thickening, Lesions and Masses)—What Can Be Seen on Transthoracic Ultrasound?

**DOI:** 10.3390/diagnostics15020176

**Published:** 2025-01-14

**Authors:** Kathleen Möller, Tomas Maruskin, Michael Ludwig, Wolfgang Blank, Stephan Eisenmann, Christian Jenssen, Hajo Findeisen, Burkhard Möller, Christoph F. Dietrich

**Affiliations:** 1 Medical Department I/Gastroenterology, SANA Hospital Lichtenberg, 10365 Berlin, Germany; k.moeller@live.de; 2 Department General Internal Medicine (DAIM), Hospitals Hirslanden Bern Beau Site, Salem and Permanence, 3013 Bern, Switzerland; tomas.maruskin@hirslanden.ch; 3 Department for Internal Medicine, Hospital of the German Armed Forces, 10115 Berlin, Germany; michael6ludwig@bundeswehr.org; 4 Medical Department I, Hospital on Steinenberg Reutlingen, 72764 Reutlingen, Germany; wolfgang.blank@icloud.com; 5 Department for Internal Medicine I, University Hospital Halle, 06120 Halle, Germany; stephan.eisenmann@uk-halle.de; 6 Department for Internal Medicine, Hospital Märkisch Oderland, 15344 Strausberg, Germany; c.jenssen@khmol.de; 7 Brandenburg Institute for Clinical Ultrasound (BICUS) at Brandenburg Medical University, 16816 Neuruppin, Germany; 8 Department for Internal Medicine, Red Cross Hospital Bremen, 28199 Bremen, Germany; findeisen.h@roteskreuzkrankenhaus.de; 9 Department of Rheumatology and Immunology, Bern University Hospital, Inselspital, University of Bern, 3010 Bern, Switzerland; burkhard.moeller@insel.ch

**Keywords:** benign pleural tumors, pleural lesions, benign tumorlike conditions, transthoracic ultrasonography, contrast-enhanced ultrasonography

## Abstract

Pleural thickening can be the result of inflammation or infection but can also have a neoplastic origin. Depending on the clinical context, a pleural lesion or mass is often initially suspected of malignancy. Benign pleural tumors are rare, and their appearance on ultrasound (US) is also described less frequently than pleural metastases or malignancies. There are few descriptions of contrast-enhanced Ultrasound (CEUS) in particular. This review introduces the basics of transthoracic ultrasound (TUS) of the pleura and CEUS of the pleura and lung. CEUS is recommended for pulmonary applications in the EFSUMB guidelines in non-hepatic applications. This article provides an overview of the characteristics of benign pleural thickening, tumor-like lesions, and benign pleural tumors on transthoracic B-mode US with color Doppler imaging (CDI) and CEUS. In detail, characteristics in TUS and CEUS are described for infectious/inflammatory pleural thickening (empyema, tuberculous pleuritis, hemothorax, fibrothorax), pleural thickening in various systemic diseases, in tumor-like conditions (plaques, splenosis, endometriosis, mesothelial cysts, lymphangiomatosis) and benign tumors (lipoma, benign SFT, schwannoma, solitary extramedullary/extraosseous plasmacytoma). The descriptions are illustrated by corresponding US and CEUS images.

## 1. Introduction

**Figure 1:**
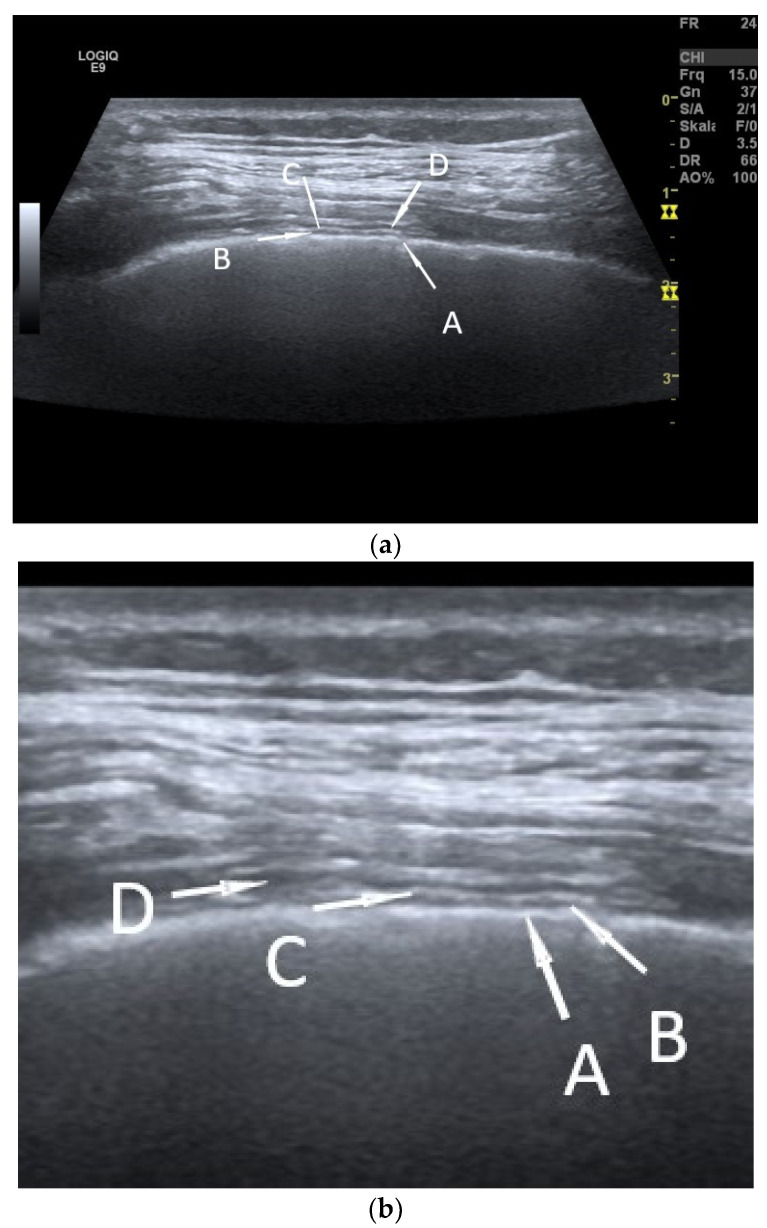
Normal pleura on high resolution TUS. The thoracic wall with the pleural structures is shown with the high-resolution linear transducer (**a**), and the pleural structures are enlarged in section (**b**). A: Total reflection of the lung and visceral pleura as hyperechoic interface echoes. B: hypoechoic interpleural space. C: parietal pleura as hyperechoic interface echo. D: hypoechoic extra pleural fat lamella.

The pleura is a hair-thin structure that consists of two layers: the visceral pleura, which envelops the lungs, and the parietal pleura, which coats the inside of the chest cavity. Both pleural layers are covered with single-lined mesothelium. Between these two pleural layers, the pleural cavity, as a usually narrow space, is filled with a very small amount of pleural fluid, leading to the cohesion of the lung to the chest wall and making the lung sliding possible, which reduces breath work. In transthoracic ultrasound (TUS) with high-frequency linear transducers, these thin pleural structures can be visualized. The pleural layers appear as hyperechoic interface echoes. The pleural space between the two pleural layers is hypoechoic [Figure 1] [1].

**Figure 2:**
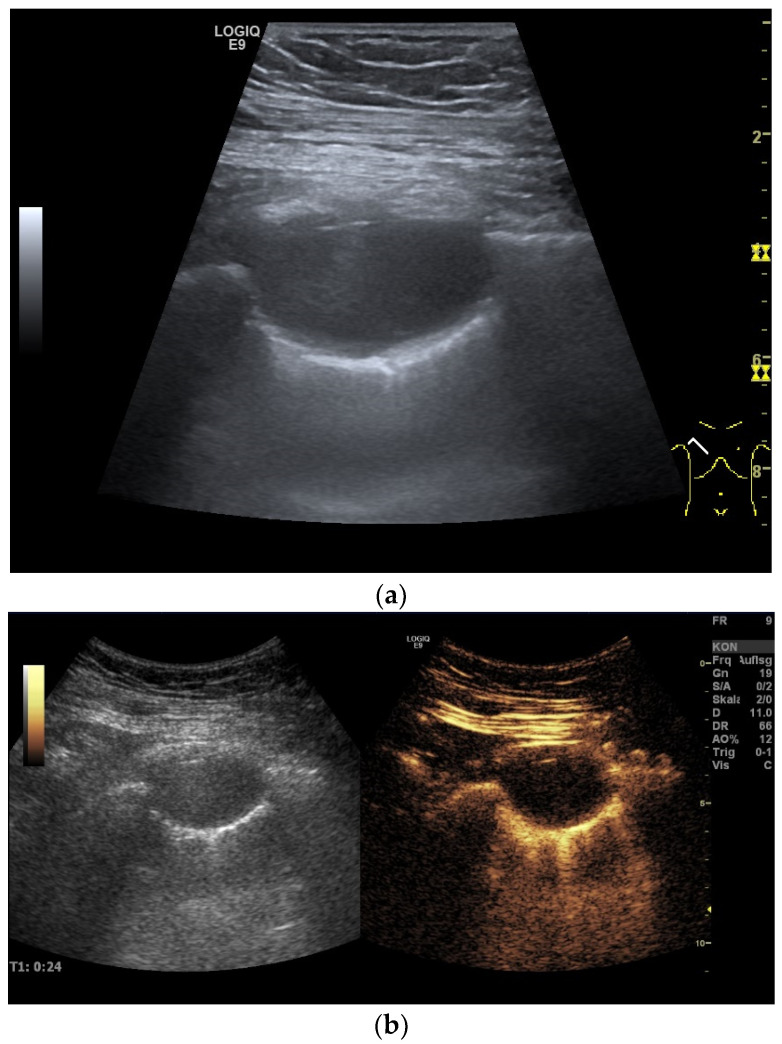
Encapsulated pleural effusion. A few months previously, a mitral valve replacement had been performed, which was accompanied by severe complications. The complications included a total right pleural effusion. No pulmonary mass was described preoperatively. In the case of pneumonia, a chest CT was performed, which showed a pleural mass in the right upper lobe. Initially, no distinction was made between a solid lesion and an encapsulated effusion, and image-guided sampling was recommended. A 38 × 24 mm hypoechoic to non-echoic well-circumscribed oval lesion in the right upper lung on high-resolution linear B-mode-ultrasound (**a**). The lesion did not show any enhancement in the CEUS at any time (**b**). A solid malignant tumor could, therefore, be excluded. In the US, the findings were, therefore, most likely attributed to an accumulation of fluid, an encapsulated effusion. The lesion was observed during the follow-up examination and regressed spontaneously over time, which was attributed to an encapsulated effusion.

Tumors of the pleura either originate primarily from the pleura or are secondary in the case of metastasis. In the case of an inflammatory disease of the lungs, the pleura can be involved. Diseases that affect the pleura commonly present with unilateral or bilateral pleural thickening and/or pleural effusion. Pleural thickening usually appears hypoechoic on ultrasound and may be diffuse or focal depending on the underlying disease. Pleural effusions usually appear non-echoic, but they can also contain small and swirling internal echoes (“swirling sign”), fibrin threads, or septa. In addition, a distinction must be made between freely leaking and encapsulated effusions. Solid lesions must be differentiated from encapsulated effusions, empyema, and necroses. A precise US description of these solid and fluid pleural changes can help in the differential diagnosis [Figure 2]. A description of various pleural lesions, masses, and thickening is summarized in Table 1.

### Advances and Limitations of TUS

The ventilated lung filled with air is a strong reflector and leads to a total reflection of the US. Reverberations with A-lines on the US are typical of the ventilated lung. B-lines occur in various diseases. Structures that are localized behind the total reflection cannot be assessed in the US under these conditions. TUS can only visualize structures that are not covered by lung artifacts. These are lesions of the pleura, peripheral subpleural lung lesions, internal structures in pleural effusions, and lesions in the atelectatic lung. Centrally located lung tumors are only seen in the TUS if the lung tissue in the ultrasound pathway is totally atelectatic.

While computed tomography (CT) scan is the gold standard in imaging of pleural and pulmonary diseases, due to its better overview and the additional imaging of both bony structures and lung parenchyma, US can provide additional diagnostic benefits as a complementary imaging modality. Although US can only be used to visualize about two-thirds of the pleural area, US also offers great additional advantages, such as very high spatial resolution and real-time imaging of movements in the lung, diaphragm, and chest wall as well as of the intravascular blood flow.

High-resolution US can distinguish between structures of the pleura and lung and can also detect subpleural lesions. The breath-dependent mobility of the lesion, its vascularization, and its contrast-enhancement patterns in contrast-enhanced ultrasound (CEUS) can also be used as additional criteria. The most important advantage of the US in terms of distinguishing pleural from pulmonary lesions is real-time imaging. If the lung slides over the lesion, it is a pleural lesion [Video S1]; if the lesion moves with the lung sliding, the lesion is of pulmonary origin [1,2,3,4,5,6]. Limitations exist in the mediastinum and in the attenuation of the ribs. In these locations, the pleura cannot be assessed on TUS. On CEUS, vascularized and non-vascularized lesions and necroses can be distinguished. Tumors, abscesses, and peripheral lung infarcts can be differentiated [7]. The intensity of the enhancement allows conclusions to be drawn about the degree of vascularization. The early enhancement of lesions before organs of the systemic circulation are typical of pulmonary artery origin. Enhancement at the same time as organs with systemic vascularization indicates bronchial arterial enhancement. The vascular supply of the parietal pleura comes from the intercostal arteries via systemic circulation. The performance of CEUS with the US contrast agent SonoVue^®^ (Lumason^®^) (Bracco Suisse SA, Geneva, Switzerland) with pulmonary and pleural indications is off-label use. The performance of CEUS is independent of kidney and thyroid function. Adverse effects are very rare. In over >463,000 patients, only 0.001% serious adverse effects and 0.034% non-serious adverse effects were reported [8]. CEUS can only assess lesions that are also visualized in TUS. What cannot be seen in the TUS cannot be assessed with CEUS either.

The aim of this review is to describe the appearance of various benign pleural thickenings, benign tumor-like conditions, and benign pleural tumors in transthoracic US and CEUS. Reference is made to malignant pleural thickenings in differential diagnosis. Regarding malignant pleural diseases on TUS and CEUS, we refer to the relevant literature and a paper from this series [9]. Our review should assist in the better integration of TUS and CEUS in the presentation and differential diagnosis of benign pleural lesions and masses.

## 2. Pleural Thickening

Malignant pleural diseases usually exhibit more pronounced thickening than benign diseases. Highly suspicious for malignancy are pleural thickness of more than 10 mm, nodularity of the pleura, and diaphragmatic thickening > 7 mm [10]. Further studies have confirmed that malignant pleural thickenings are more pronounced than benign ones. Benign pleural thickenings were mostly uniform, whereas malignant ones were both uniform and wavy/papillary or masses [11].

In a study by Findeisen et al. with lesions of the parietal pleura, the pleura was generally significantly thickened. However, the benign parietal pleural lesions were narrower than the malignant ones [12].

While the lung in CEUS shows an enhancement pattern via the pulmonary arteries and bronchial arteries, parietal pleural lesions reveal a systemic vascularization pattern via the intercostal arteries. Malignant lesions of the parietal pleura were significantly more perfused semi-quantitatively than benign lesions with a predominantly reduced/absent perfusion. However, there were also benign parietal pleura lesions with marked perfusion [12].

In the study of Yang et al. on CEUS, the arrival time and the time to peak of TIC were longer in benign pleural thickening than in malignant pleural thickening. The cutoff of the arrival time was 16.8 s, with a sensitivity of 83.3% and specificity of 65.0% for malignancy. It could, therefore, be concluded that these parameters in CEUS alone are not reliable in the differential diagnosis between benign and malignant lesions in pleural lesions. However, the intensity of contrast enhancement was higher in malignant pleural thickening than in benign pleural thickening. In combination with the extent and sonomorphology of pleural thickening and CEUS parameters, it was possible to differentiate between benign and malignant wall thickening with sensitivity and specificity of 93.3% and 90.0%, respectively [11].

US-guided sampling is the method of choice in unclear wall thickening and lesions to obtain histologically evaluable material [13]. CEUS can be helpful in distinguishing vascularized solid tissue from non-vascularized necrosis [14,15].

Thoracoscopy is indicated for the assessment of pleural effusions of unknown etiology and pleural thickening for histologic diagnosis and staging of malignant pleural diseases [16].

## 3. Infectious and Inflammatory, Post-traumatic

### 3.1. Empyema

**Figure 3:**
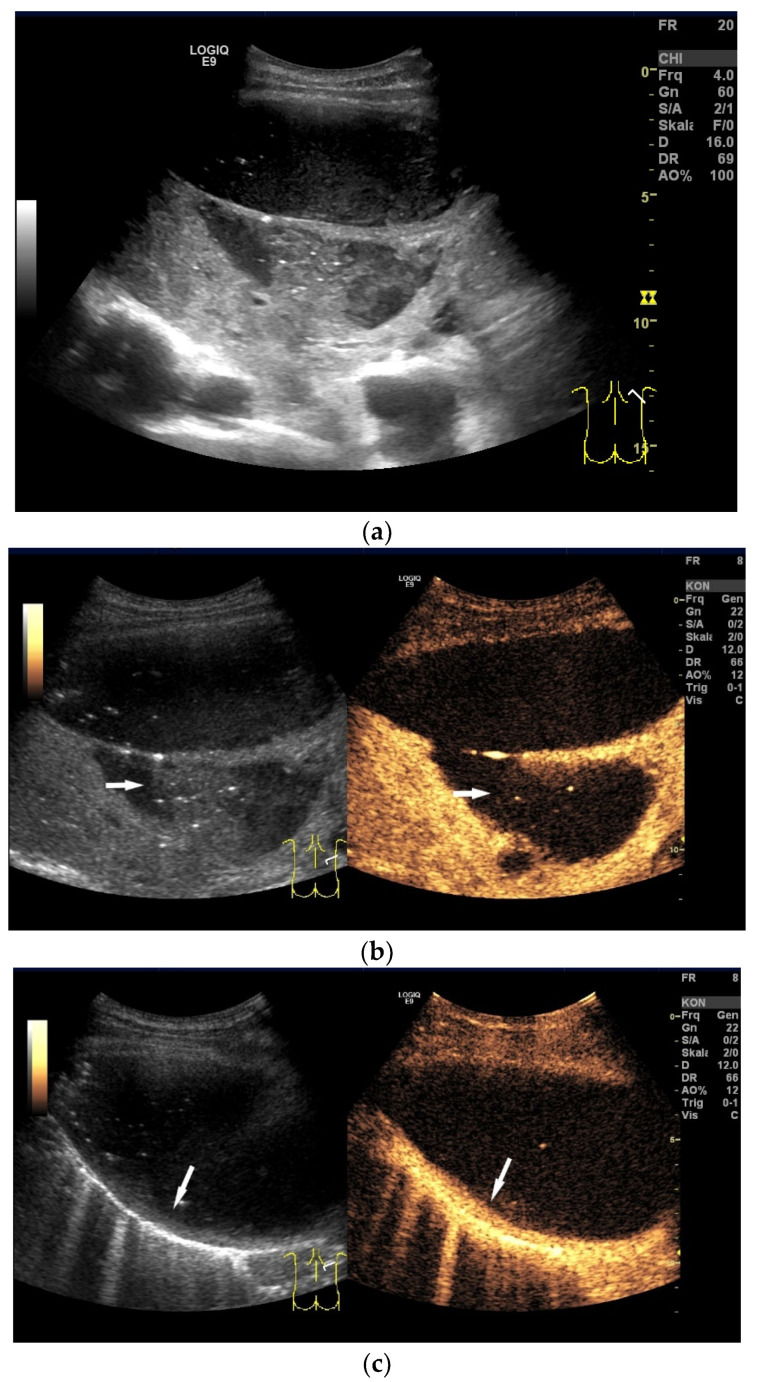
Pleural empyema, pneumonia with pleural effusion, sepsis. Pleural effusion with hypoechoic inhomogeneous internal reflexes and echogenic air reflexes (**a**). In CEUS, the hypoechoic internal structures are nonenhanced (arrow), i.e., not solid but corresponding to thickened fluid (**b**). In another transducer position, the empyema is encapsulated, and the pleura is thickened and strongly enhanced on CEUS (arrow) (**c**).

**Figure 4:**
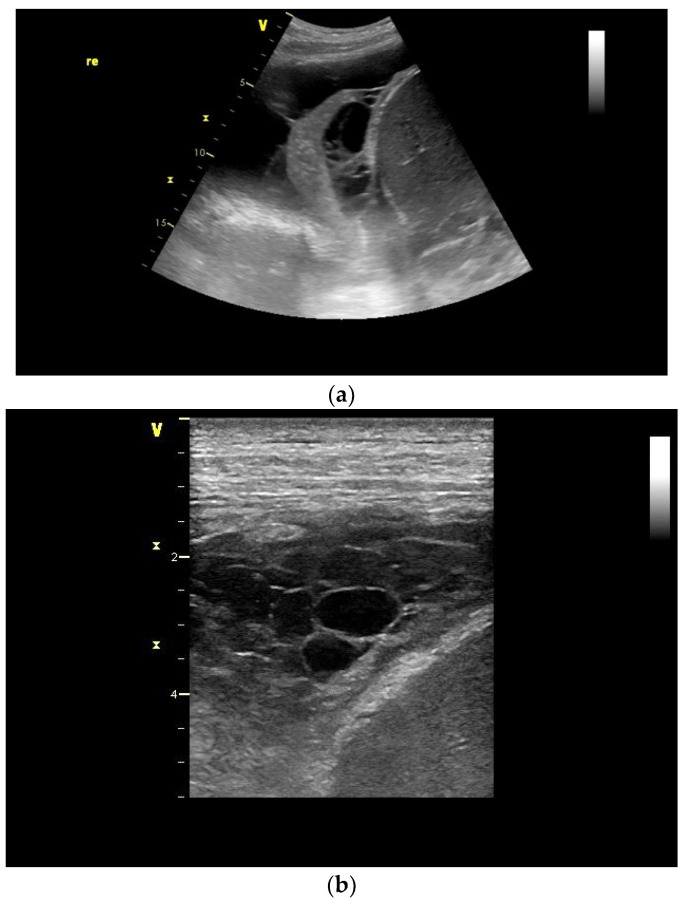
Septate pleural empyema with many fibrin strands between the atelectatic lung and pleura (**a**) and formation of a strong chambering with clearly thickened fibrosed hypoechoic pleura (**b**). re—right thoracic hemispheres.

Empyema can develop as a complication of pneumonia after thoracic trauma, thoracic surgery, esophageal perforation, or after esophageal surgery. The patients show severe signs of illness. An empyema goes through several stages: first, the exudative phase, then the purulent phase, and finally, the organization phase. In the exudative stage, fibrin strands and floating echoes appear, and the effusion can be septate and of varying echogenicity. In the second fibrinopurulent stage, the pleura is thickened due to fibrin deposits. In the last organizing phase, a rigid, thick pleura forms around the length [17,18,19,20,21] [Figure 3 and Figure 4].

Pus is hypoechoic. The pleural cavity can be chambered due to fibrin. As a result of the acute inflammation, the pleura is hypoechoic and thickened. Air pockets in the pleural cavity can either be caused by anaerobic pathogens or be traced back to a bronchopulmonary fistula [17].

It is generally important in any type of imaging to differentiate between an empyema and a peripheral lung abscess. A lung abscess is usually treated with long-term antibiotic therapy and usually conservative measures, whereas an empyema usually requires percutaneous or surgical drainage in combination with antibiotics.

**Figure 5:**
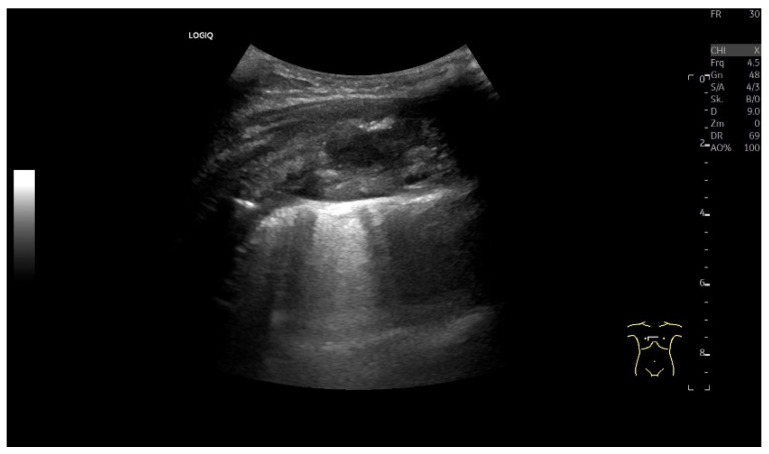
Pleural thickening and thoracic wall destruction after empyema necessitans . Patient after surgery for cardiac carcinoma with complications including anastomotic insufficiency, mediastinal abscess, and pleural abscess. Endovac (endoscopic vacuum) therapy and percutaneous abscess drainage were performed. Development of swelling of the thoracic wall during the course. In the TUS, inhomogeneous chest wall, hypoechoic lesions, and thickened parietal pleura are demonstrated . Under suspicion of metastasis, a US-guided biopsy was performed several times. This revealed granulomatous inflammation and no evidence of a tumor.

A special form is ***empyema necessitans***. This is assumed when the empyema exceeds the parietal pleura and infiltrates the surrounding soft tissue and chest wall muscles [22,23,24,25,26]. Neighboring organs can also be infiltrated. Imaging shows a well-defined fluid collection that exceeds the pleura [Figure 5].

An important differential diagnosis of pleural empyema is peripheral subpleural lung abscesses. These are usually surrounded by a thick capsule. Internal structures in pleural empyema and lung abscesses are not enhanced in CEUS.

### 3.2. Tuberculous Pleuritis

**Figure 6:**
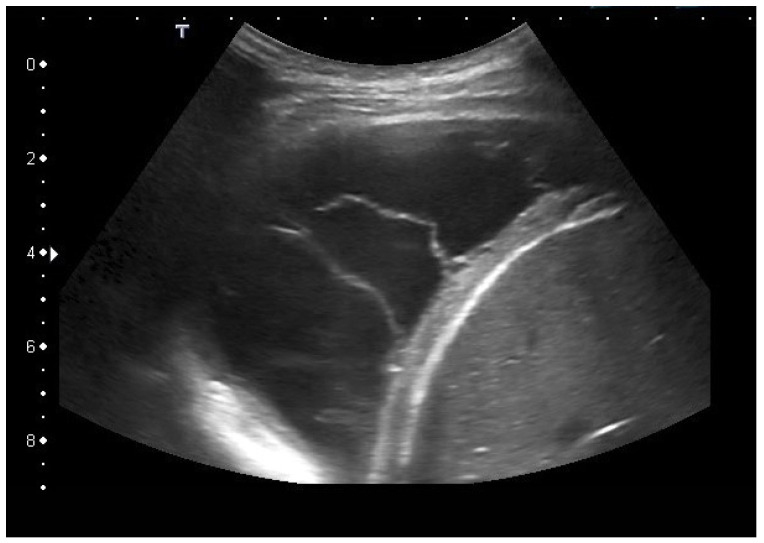
Pleural manifestation of tuberculosis with pleural effusion, markedly thickened hypoechoic pleura, and fibrin strands.

**Figure 7:**
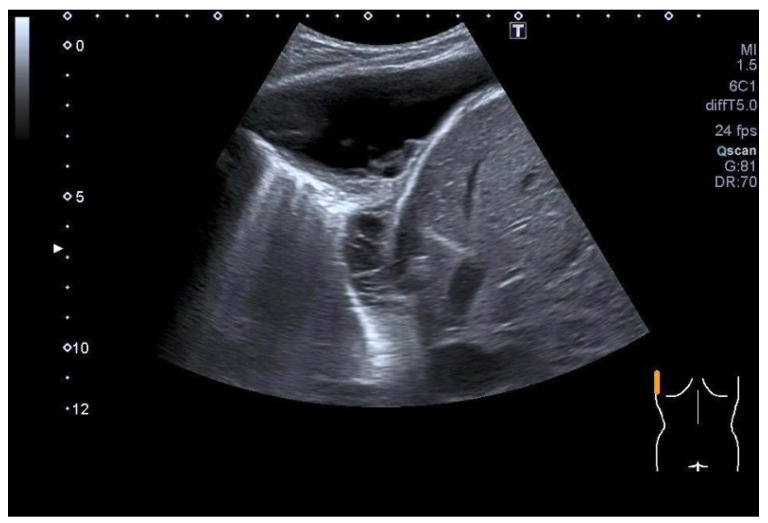
Tuberculous pleurisy with pleural effusion, irregular small pleural thickening, and complex septation.

In tuberculosis, pleural effusion, pleural thickening, and pleural calcification are of interest and diagnostically relevant [Figure 6]. In the pleural effusion, echogenic strands and complex septations appear [27,28,29,30] [Figure 7]. In a study, pleural effusion and pleural calcifications were diagnosed more accurately on TUS than on chest CT. However, CT was more sensitive for the detection of pleural thickening. While the sensitivity of ultrasound for the diagnosis of pleural effusions (78%) and pleural calcifications (74%) was comparable to CT (71.8% and 72.6%), ultrasound was significantly worse in the detection of pleural thickening. If CT is taken as the “gold standard”, 21.8% of patients had pleural thickening on CT, while only 2.8% of the same patient selections had pleural thickening on US. [28].

Granulomatous inflammation and the formation of granulomas are typical of tuberculosis. These are initially ill-defined and then round in the B-mode hypoechoic. On CEUS, both are hyper-enhanced. If caseous necrosis develops, these are hypoechoic lesions in B-mode US, in CEUS hypo- or non-enhanced, heterogeneously enhanced lesions, with contrast-enhanced septations and contrast-enhanced rim [30]. These lesions should be searched for in the thickened pleura. CEUS can be beneficial in US-guided biopsy [31].

### 3.3. Hemothorax

**Figure 8:**
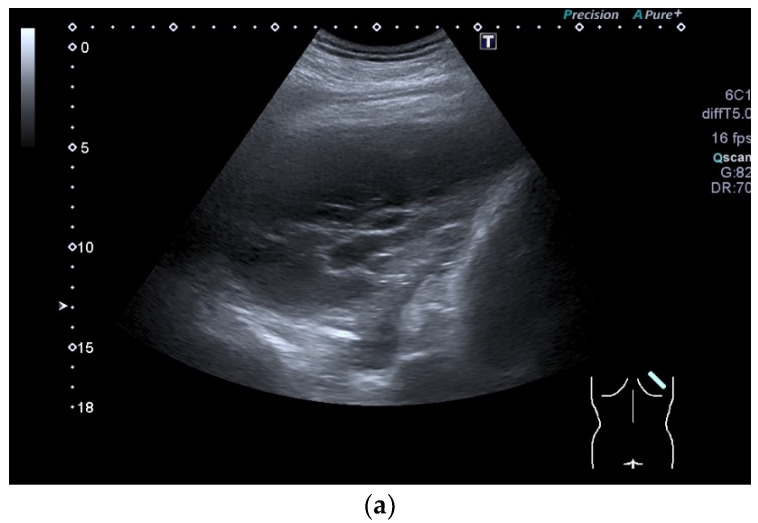

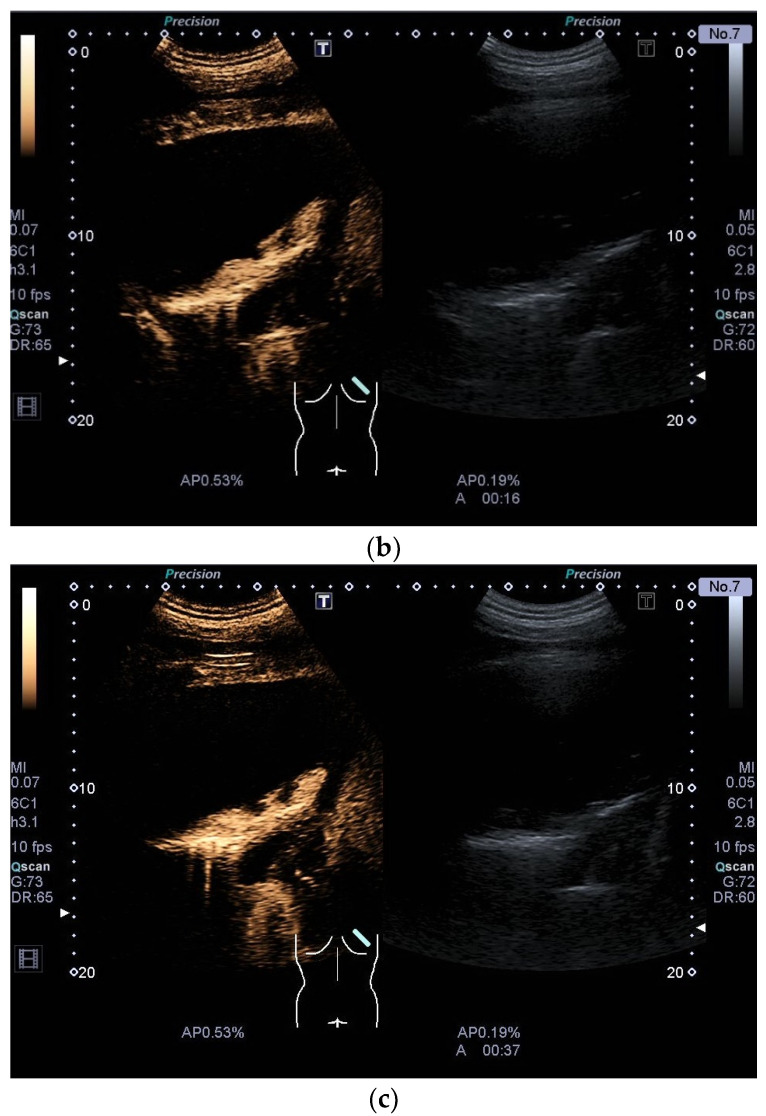
Hemothorax. Road traffic accident with rib fracture and hemothorax. The TUS shows a pleural effusion with inhomogeneous internal structures (**a**). On CEUS, after 16 s and 37 s, the atelectatic lung tissue is enhanced (left image side). The remaining nonenhanced structures in the effusion correspond to the blood coagulum (**b**,**c**).

**Figure 9:**
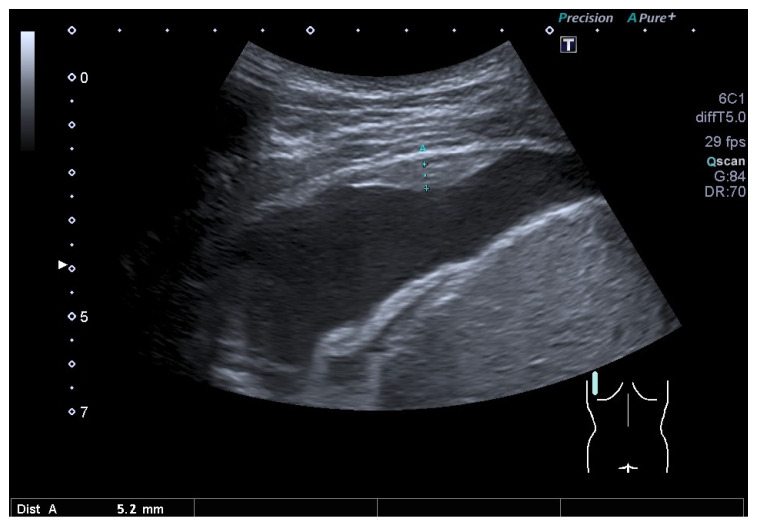
Hemothorax one week after a bicycle accident with a rib fracture. A young man with no history of tumors. Hypoechoic pleural effusion and focal pleural thickening (between the markers). Dist A—distance A.

Hemothorax occurs post-traumatically after accidents but also after interventions on the thorax. The pleural effusion is usually not non-echoic but with internal contrast or hypoechoic content [Figure 8]. After some days, a hemothorax usually becomes septate and/or encapsulated. The pleura can thicken [Figure 9] and show calcifications. In non-traumatic catamenial hemothorax, especially with pneumothorax and small pleural nodules, endometriosis should be considered [32].

### 3.4. Fibrothorax

**Figure 10:**
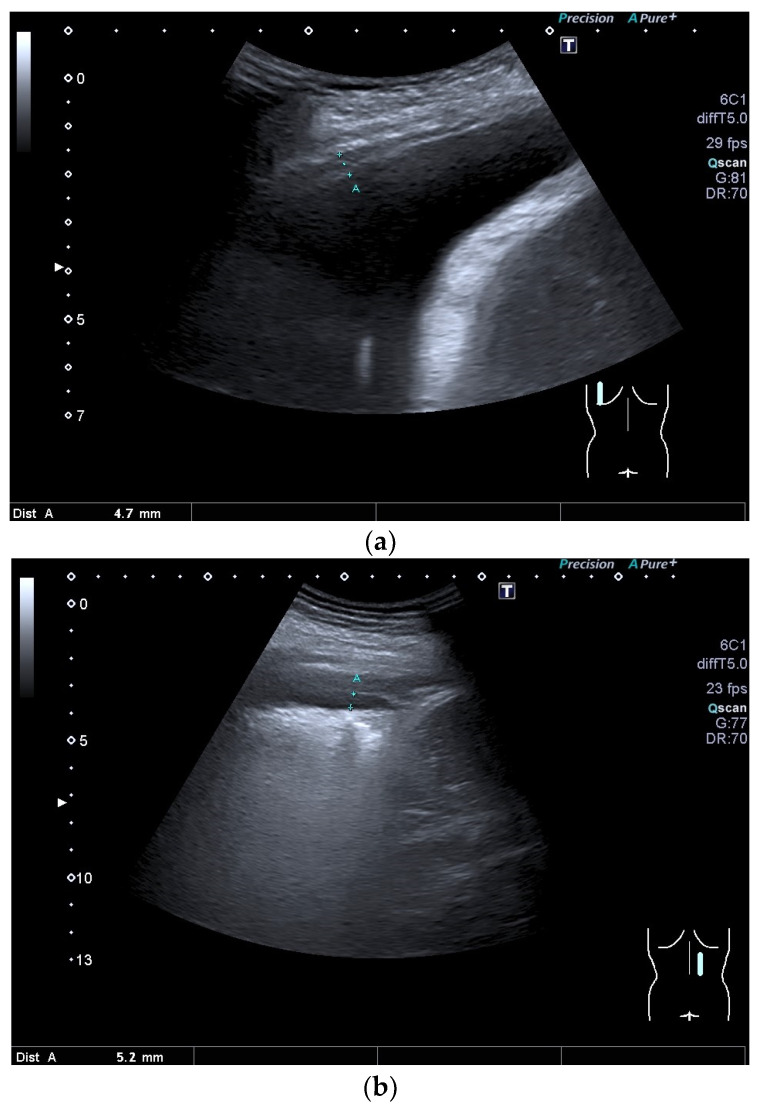
Development of hypoechoic pleural thickening in a recurrent post-inflammatory pleural effusion (between the markers). The pleura is clearly hyperechoic and thickened above the diaphragm (**a**). After the effusion has subsided, the hypoechoic thickening of the pleura remains. Between the markers, there is still a narrow amount of fluid in the pleural cavity (**b**). This suggests that the pleural slide is still preserved. Dist A—distance A.

Fibrothorax corresponds to the most severe form of pleural fibrosis. This leads to extensive and dense fibrosis of the visceral pleura, with fusion of the visceral and parietal pleural layers [33,34]. It is a condition that follows previous pleurisy of various origins [32]. The most frequent causes are asbestos exposure, tuberculosis, empyema, and hemothorax. If the parietal pleura is thickened and shows calcifications, there is a suspicion of asbestos-induced fibrothorax if there is a history of asbestos exposure. On TUS, it presents as a uniformly thin hypoechoic layer (<1 cm) and no lung sliding [Figure 10].

## 4. Systemic Diseases

### 4.1. IgG4 Related Diseases

IgG4-associated diseases are systemic diseases involving various organs due to fibroinflammatory lesions with dense lymphoplasmacytic infiltration, storiform-type fibrosis, obliterative phlebitis, pronounced infiltration of lymphocytes, and IgG4-positive plasma cells [35]. Serum IgG4 levels should be elevated at least 2–3 times. Middle-aged to elderly males with a median age of diagnosis of 60 years are mostly affected [36,37]. The most common manifestations are the hepatobiliary system, pancreas, retroperitoneum, lymph nodes, salivary and lacrimal glands. All organs, including the lungs, can be affected in about 14% of cases. The pleura is rarely affected [35]. In a pretherapeutic CT study of 48 patients with thoracic involvement, only 8% of them had pleural manifestation [38]. Pleural manifestations include pleural nodules, pleural thickening, and pleural effusion [37,39]. Other intrathoracic manifestations include hilar and mediastinal lymphadenopathy, pulmonary infiltrations, thickening of broncho vascular bundles, and interlobular septa [38,40,41,42]. 43% of the patients with IgG4-related lung and pleural disease had extrathoracic lesions simultaneously or asynchronously during follow-up [36].

Iijima et al. reported on smoothly circumscribed round and oval pleural nodules measuring up to 29 mm [39]. Kim et al. reported a solitary flat oval pleura-based mass of a maximum of 30 mm [43]. The nodules can relate to both the parietal and visceral pleura and extend into the subpleural tissue and the lungs [36].

These nodules showed a heterogeneous pale contrast on CT [39].

### 4.2. Sarcoidosis

Sarcoidosis is a multisystem disease characterized by non-caseating granulomas. Typical manifestations are the enlargement of mediastinal and bihilar lymph nodes and nodular pulmonal lesions. But all other organs can be affected. Nevertheless, pleural involvement seems to be rare. Pleural manifestation may include pleural effusion, pneumothorax, pleural plaques, pleural thickening, and nodules [44]. Hydropneumothorax, hemothorax or chylothorax are even rarer [45]. Pleural effusions caused by sarcoidosis are typically exudative and show an increased number of lymphocytes [46].

In 1974, Chusid and Siltzbach reported that only 0.9% of 2410 patients with sarcoidosis had pleural manifestations [47]. 18/22 were histologically confirmed. Otherwise, exudative pleural effusions with increased lymphocytes were the basis for diagnosis when the underlying disease was confirmed. It was suspected that the number of unreported cases was higher [47]. In a systematic outpatient TUS of 181 sarcoidosis patients, 2.8% had a pleural effusion. After excluding other causes, 1.2% were found to be due to sarcoidosis [48].

However, thickening of the pleural surface in 25 patients with sarcoidosis has been reported in up to 20% of CT scans [49]. In a study using HR-CT on 61 selected sarcoidosis patients, 41% of cases involved the pleura. This included 20 cases of pleural thickening and 5 cases of pleural effusion. Bilateral pleural thickening was more frequently observed in patients with CT findings of lung parenchymal fibrosis [50]. However, it has been discussed that retraction of the pleura and extrathoracic soft tissue associated with fibrotic pulmonary sarcoidosis mimics pleural thickening.

Thoracoscopy (thoracotomy) revealed multiple very small white nodules [47,51]. However, Sunnetciouglu et al. reported massive pleural lesions bilaterally and in the areas of pleural thickening on CT [44].

Subpleural nodules are common in HR-CT in sarcoidosis. And subpleural lung nodules can indent the pleura, unlike pleural nodules [45].

Safai Zadeh et al. describe the perfusion patterns of peripheral pulmonary granulomatous lesions (PPGLs) with various causes by contrast-enhanced ultrasound (CEUS) [6]. However, regarding the pleura, we are not aware of any TUS B-mode or CEUS studies describing sarcoidosis-specific B-mode findings of the pleura.

### 4.3. Amyloidosis

Thoracic amyloidosis can manifest in the lungs, bronchi, mediastinum, and pleura. Pleural manifestation is rare and accounts for only 1–2% of patients with systemic amyloidosis [52]. Bilateral pleural effusions, diffuse pleural thickening, pleural plaques, or mass-like pleural thickening with progressive enlargement are possible manifestations [52,53]. Calcifications are possible [54]. US-guided sampling and histopathological processing with Congo red are possible for diagnosis [52].

### 4.4. Miscellaneous

Horn et al. demonstrated TUS images of various pleural lesions such as scarring, scleroderma, GvHD of the lung, tuberculosis, Mediterranean fever, and neurofibroma [1]. All lesions were uncharacteristic and nonspecific on B-mode US. The authors emphasized that clinical context is essential [1].

*Connective tissue diseases* such as rheumatoid arthritis or systemic lupus erythematosus and *vasculitis diseases* such as granulomatosis with polyangiitis (Wegener’s granulomatosis) or eosinophilic granulomatosis with polyangiitis (Churg Strauss syndrome) very rarely have pleural involvement (approx. 1%). This is usually a pleural effusion. Pleural thickening has also been described in general but without any specifics [55].

## 5. Tumor-like Lesions

### 5.1. Benign Asbestos-Related Diseases

Long-term exposure to asbestos can lead to pulmonary fibrosis (asbestosis) and (malignant) mesothelioma of the pleura, but also to benign pleural diseases. These include pleural plaques, benign asbestos pleural effusion (BAPE), diffuse pleural thickening (DPT), and rounded atelectasis [32,56,57].

***Plaques*** are flat or nodular pleural hyaline thickenings; they usually originate from the parietal pleura, only rarely from the visceral layer. On ultrasound, pleural plaques appear as ovoid, hypoechoic, homogeneous lesions [17]. They may exhibit calcifications. Typical localizations of pleural plaques are posterolateral, anterolateral, in the area of the diaphragmatic dome, and mediastinal. The lung apex and the costodiaphragmatic angle are usually not affected. The pleura in the area of the mediastinum is not assessable on TUS. The localization in the area of the lung apex and in the costodiaphragmatic angle is usually not affected. Bilateral calcified pleural plaques are usually due to previous exposure to asbestos. However, other non-asbestos-related differential diagnoses must also be considered. These include previous tuberculous pleurisy, hemothorax, talc pleurodesis, and early mesothelioma [32,56].

***Diffuse pleural thickening (DPT)*** affects the visceral pleura but can also include the parietal pleura. The pleura may be markedly thickened and envelop the lung or may be thickened only to a limited extent but involve at least a quarter of the thoracic wall. An important differential diagnosis is malignant mesothelioma. Diffuse pleural thickening may be associated with a benign pleural effusion. The differential diagnosis between benign pleural thickening and mesothelioma can be difficult and may require a thoracoscopic biopsy [32,56].

In the case of ***rounded atelectasis (or Blesovsky syndrome)***, a supposed mass develops. Focal inflammation of the pleura leads to a fusion of the parietal and visceral pleura with thickening. The adjacent lung tissue is compressed and collapses [32,56].

In CEUS, atelectatic lung tissue is characterized by early pulmonary arterial vascularization, while tumors show later bronchial arterial vascularization [58].

### 5.2. Thoracic Splenosis

Thoracic or pleural splenoses occur due to the spread of splenic tissue during trauma and postoperatively. Intrathoracic and pleural splenoses were seen primarily on the left side and were associated with diaphragmatic injury. In the constellation of trauma, splenectomy, and diaphragmatic injury, the prevalence of intrathoracic splenosis is given as 18% [59]. Medical history can be indicative. Splenoses are asymptomatic and benign but may have differential diagnostic significance in distinguishing them from other masses or tumors.

Föh et al. describe multiple intra-abdominal and intrathoracic splenosis after splenectomy [60]. This was also diagnosed using CEUS, and long-term contrast enhancement, and a biopsy was avoided [60]. Kroenig et al. describe a pleural splenosis in the left thorax, which was diagnosed by CEUS and confirmed by US-guided sampling with histological examination [61]. The lesion was smoothly bordered, round, and homogeneously hypoechoic. In CEUS, the lesion showed systemic arterial enhancement with persistence of the strong enhancement even after 6 min [61]. The long-lasting enhancement over several minutes is a typical characteristic of splenic tissue [7].

In general, it must be distinguished between accessory spleen and splenosis. The accessory spleen is a congenital condition that results from a failed fusion of the splenic tissue during embryonic development. Accessory spleens are usually round, well-encapsulated, contain a normal vascular hilum within normal splenic tissue, and are supplied by small branches of the splenic artery. Splenosis is unencapsulated or poorly encapsulated, has no characteristic shape, and contains some distorted splenic tissue. Splenic tissue in splenosis usually reveals a modified architecture. Normal splenic vasculature is absent in the implanted splenic tissue, which may result in a different blood supply. Splenoses have no vascular hilum and receive their blood supply from surrounding tissues [62,63,64]. Despite the different architecture, splenoses and accessory spleens have the same behavior on CEUS as the normal spleen [7]. Xiao et al. investigated in a meta-analysis the value of CT, MRI, and CEUS in the confirmation of splenoses at different locations [65]. Only 2/22 were intrathoracic [60,61]. On CEUS, 91% of the splenosis showed arterial hyper-enhancement. In the delayed phase, only 3/22 cases showed iso-enhancement or obvious washout. CEUS had a higher consistency in detecting splenosis compared to CT and MRI in the arterial and delayed phases [65].

For the sake of completeness, it should be mentioned that 99mTcsulfa colloid radionuclide scintigraphy is specific for the detection of spleen tissue [66].

### 5.3. Diffuse Pulmonary Lymphangiomatosis (DPL)

Diffuse pulmonary lymphangiomatosis (DPL) is a rare congenital disease caused by diffuse infiltration of lymphangiomas in the thorax. It mainly affects children and young adults. Extensive pleural thickening may be present. Otherwise, the changes affect the lungs, bronchi, and mediastinum. Smooth symmetrical interlobular septal and peribronchovascular interstitial thickening, patchy ground-glass attenuation, diffuse pleura thickening, extra pleural soft-tissue thickening, mediastinal soft-tissue infiltration, pleural and pericardial effusion have been described [32].

### 5.4. Endometriosis

Endometriosis is defined as the presence of endometrial glands and stroma outside the uterine cavity. Endometriosis lesions can vary in size. Deep endometriosis differs from peritoneal (or superficial) and ovarian endometriosis by the presence of endometriotic nodules larger than 5 mm [67]. Deep endometriosis may be localized in extrapelvic localizations [68].

The key feature of thoracic endometriosis is the combination of characteristic catamenial (or recurrent) pneumothorax, hemothorax, catamenial chest pain, and hemoptysis [68]. This may be the result of the implantation of endometrial tissue into the pleural space, lung, and diaphragm. CT describes small contrast-enhanced soft nodules on the pleura with pneumothorax and hemothorax [54]. In a meta-analysis, 34 case series studies with 628 female patients with thoracic endometriosis were analyzed, including five studies with 90 patients with pleural endometriosis [68]. Eighty percent of the thoracic endometriosis was located on the right side. In addition, 1.1% had simultaneous manifestations in the pleura, diaphragm, and lungs, and 12.7% had isolated manifestations in the pleura [68]. In a study of 160 female patients who underwent thoracoscopy, endometriotic lesions were located equally on the visceral or parietal pleura. Both the visceral and parietal pleura may be affected. Some patients had multiple lesions. Macroscopically, cystic lesions, brown nodules/blueberry spots, and combined cases are described. [69].

MRI findings of diaphragmatic lesions varied from punctate spots and plaques to deep nodules [68]. The gold standard for the diagnosis of thoracal endometriosis is assisted video-assisted thoracoscopic surgery (VATS) [70].

There are no CEUS studies on thoracic or pleural endometriosis lesions. CEUS data from other manifestations could be used if the clinical picture is appropriate and the lesion is visible on ultrasound. In a study of seven patients with confirmed deep endometriosis lesions, the lesions were described as irregularly hypoechoic on B-mode US or heterogeneous with dotted blood flow signal on color Doppler. On CEUS, 6 of 7 DE lesions showed heterogeneous hypoenhancement in the arterial phase. All lesions then showed an early rapid heterogeneous washout beginning in the late arterial phase [71].

### 5.5. Mesothelial Cysts

Mesothelial cysts are composed of mesothelial epithelium. They derive from the peritoneum, pericard, and pleura. Intrathoracic mesothelial cysts almost always arise in the mediastinum and are derived from the pericardium. Pericardial cysts occur congenitally. Pleural mesothelial cysts are very rare. The parietal pleura is seen as the origin [72]. Their occurrence is seen in connection with inflammatory processes [72,73]. There are no ultrasound descriptions of pleural mesothelial cysts. In one case report, a mesothelial pleural cyst was 20 mm in size and lined with a single-row cuboid epithelium [73]. It can, therefore, be seen that these show cyst criteria in the TUS. The significance is unclear.

In one case report, CT showed a gradually increasing low-density mass.

MRI showed a low-intensity mass on T1-weighted imaging and a high-intensity mass on T2-weighted imaging [73].

A differential diagnosis must be performed with other cystic lesions of the pleura. These could be, for example, cystic endometriosis lesions, pulmonary echinococcus, and cystic schwannoma.

## 6. Benign Tumors

### 6.1. Lipoma

Pleural lipomas are benign fatty lesions that arise in the parietal pleura. They are encapsulated, well-demarcated, mature fatty tissue. Lipomas can extend into the subpleural, pleural, or extra-pleural space.

In US, lipomas are not hyperechoic. On TUS, a large lipoma is described as a heterogeneous hypoechoic mass without any calcification or internal vascular supply [74].

In a study on benign and malignant pleural thickening, Findeisen et al. recorded three cases of pleural lipoma. They demonstrate one case in B-mode US, which is hypoechoic, oval, and pleural consolidation. CEUS showed only a low inhomogeneous enhancement of the lesion [12].

In a dynamic contrast-enhanced (DCE-) US study with soft tissue tumors, lipomas showed low enhancement [75].

**Figure 11:**
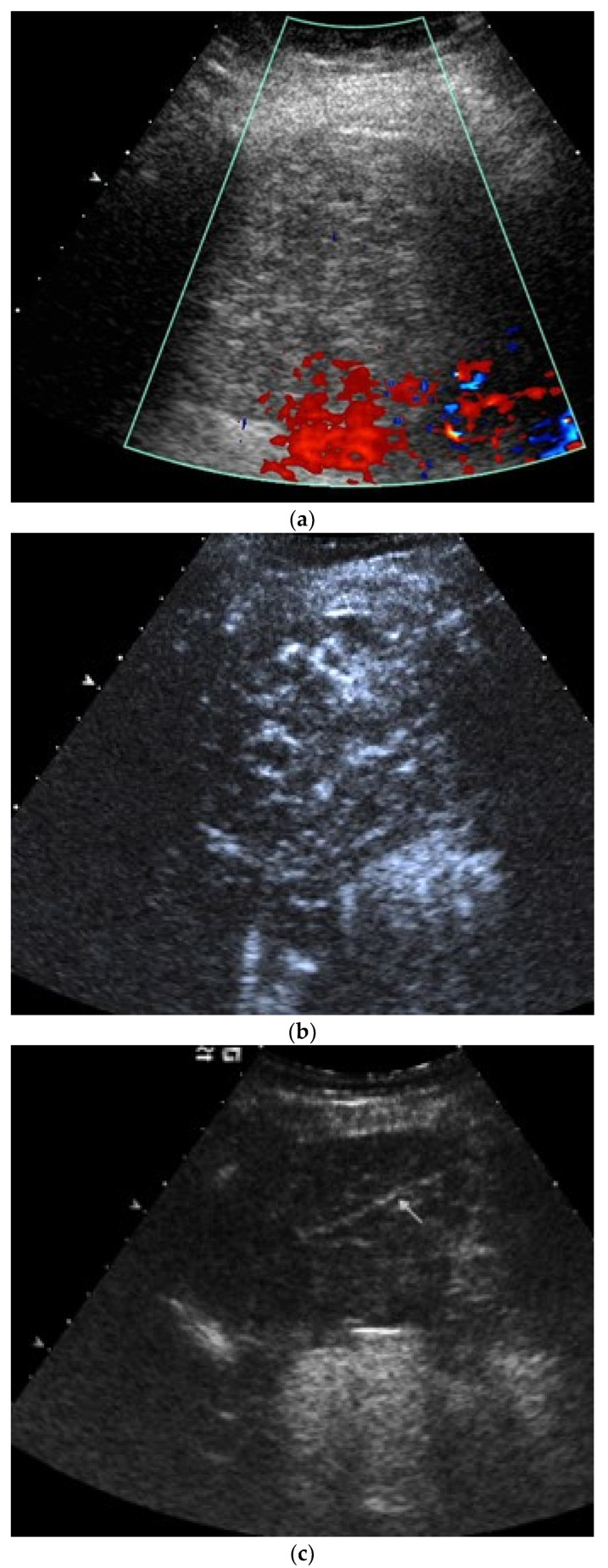
Lipoma. Female patient with a history of coughing. CT suspected pleural lipoma or liposarcoma. B-mode ultrasound and Color Doppler Imaging (**a**) revealed a hypoechoic lesion near the heart (color artifacts). CEUS showed sparse but homogenous contrast enhancement (**b**). Ultrasound-guided biopsy and histological evaluation confirmed lipoma. The biopsy needle is marked with an arrow (**c**).

On CT, lipomas are characterized by fatty attenuation (e50 to e150 HU); they are homogeneous and may have some soft tissue strands [76]. On contrast-enhanced (CE-) CT, lipomas should show no contrast enhancement and no intervening soft tissue areas within the lesion [74,76]. Otherwise, the possibility of liposarcoma must be considered [54] [Figure 11].

### 6.2. Benign Solitary Fibrous Tumor

Solitary fibrous tumor (SFT) is a mesenchymal tumor and originates from the subpleural mesenchymal cells. In a study of 223 patients, 63% were benign and 37% malignant. Two-thirds of the tumors were associated with the visceral pleura, often pedunculated. The other originated in the parietal pleura of the chest wall, diaphragm, or mediastinum [77]. A quarter of the patients had paraneoplastic hypoglycemia [77]. This is a paraneoplastic syndrome secondary to the production of insulin-like growth factor by the tumor. Further extra thoracic symptoms include pulmonary osteoarthropathy and digital clubbing [78].

In 2012, Enon et al. reported on 821 published cases, of which 736 (89%) were benign [78].

**Figure 12:**
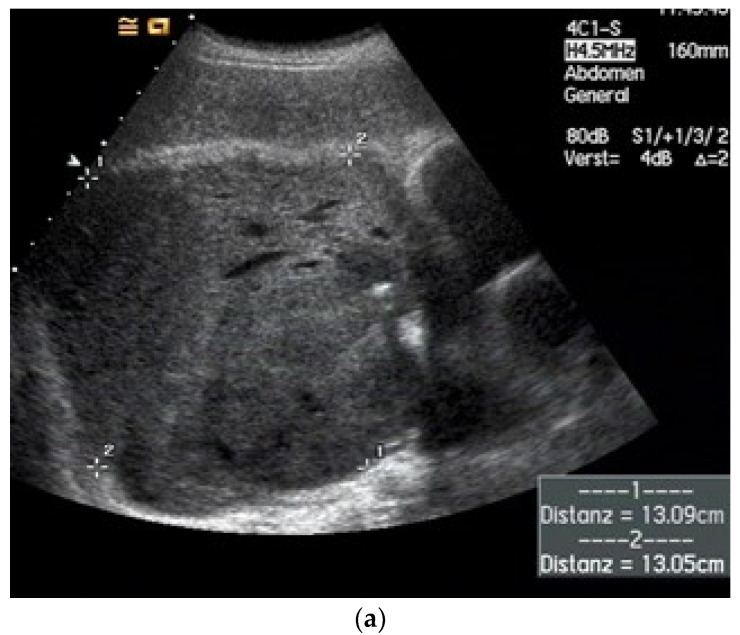

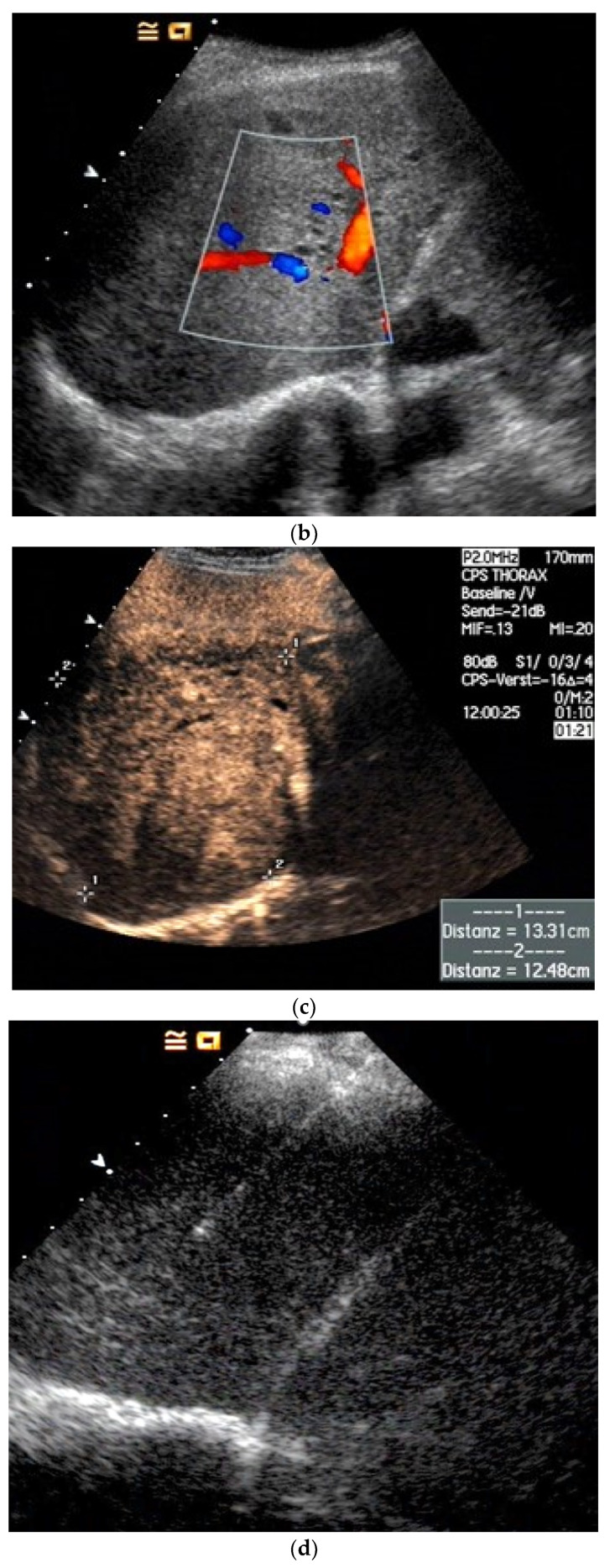
Solitary fibrous tumor of the visceral pleura. A lipomatous mass of the right pleura (8 cm) without clinical symptoms was diagnosed 2 years ago. At that time, the mass did not enhance the contrast medium on CT and was interpreted as a cyst. CEUS showed a homogeneous contrast image, albeit very discreet. US-guided transthoracic biopsy was performed. Histology revealed a mixed picture of lipomatous, mesenchymal, and lung tissue. The patient decided against surgical resection. Two years later, the patient suffered from dyspnea, and the mass showed a significant increase in size on CT. The B-mode ultrasound demonstrates a well-defined, hypoechoic tumor (diameter 13 cm) above the diaphragm. The tumor extends on the right side in the costophrenic corner to the mediastinum; there are no signs of infiltration of the lung (**a**). Color Doppler imaging detects individual large vessels in the tumor (**b**). The spectral curves were typical for bronchial arteries (not presented here). CEUS (SonoVue^®^, 2 mL) showed inhomogeneous enhancement in the bronchial arterial phase (**c**). The biopsy needle (diameter 1.2 mm) is to be monitored in B-mode US (**d**). Histology: Solitary fibrous tumor. The tumor was completely removed surgically. Final diagnosis: Solitary fibrous pleural tumor (SFPT) without signs of malignancy. There was no recurrence in the follow-up.

**Figure 13:**
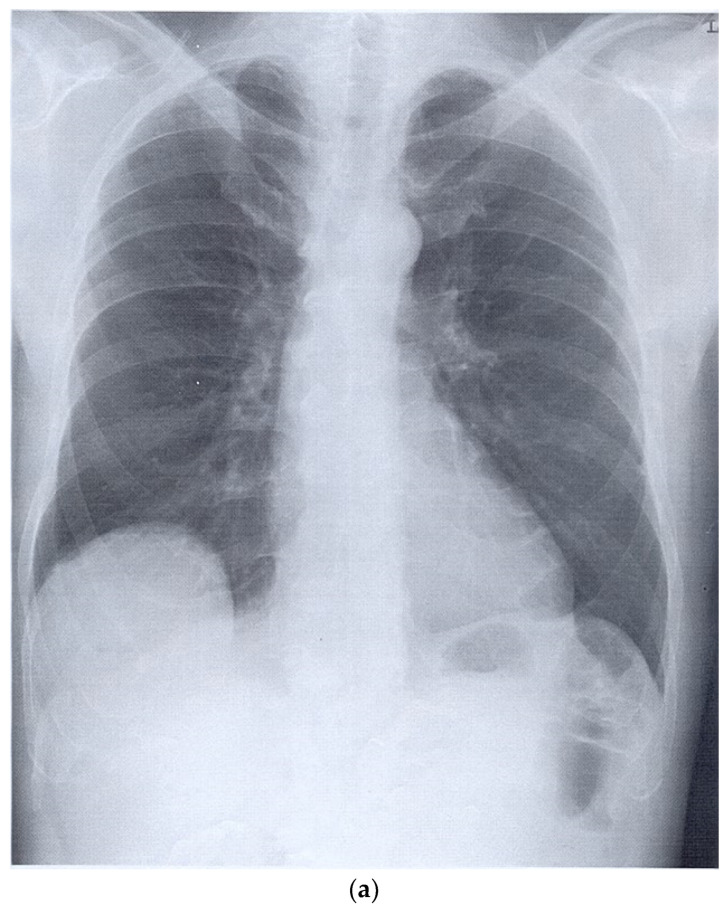

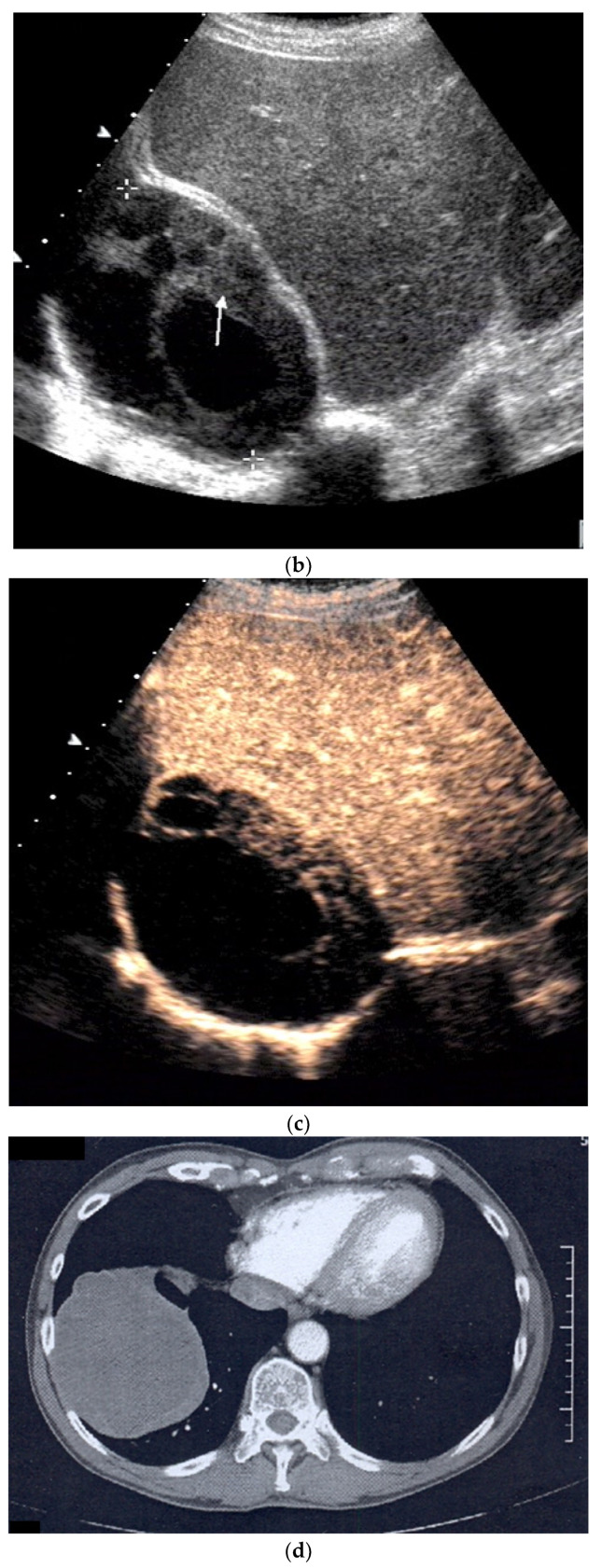

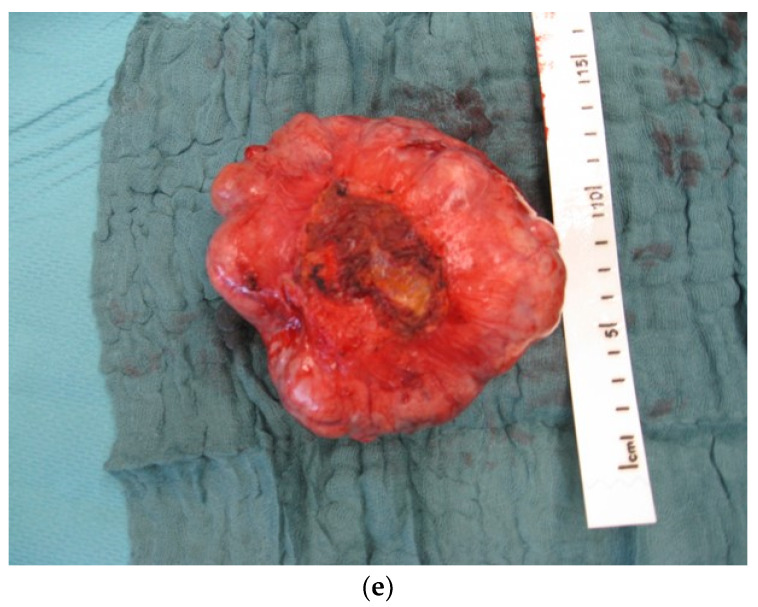
Solitary fibrous tumor of the parietal pleura of the diaphragm. In an X-ray, a mass was diagnosed adjacent to the diaphragm in the right thoracic hemisphere. (**a**). The B-mode US shows a well-defined lesion (diameter 10 cm) with solid parts (arrow) and several septa toward the diaphragm. There were no calcifications. No vessels were visible on color Doppler imaging. The lesion appears to be growing out of the parietal pleura of the diaphragm (**b**). In the dynamic B-mode US examination, normal lung sliding was visible. On CEUS (SonoVue^®^), the mass in the late bronchial arterial phase presented a slight enhancement of the solid parts in the marginal area, in the area of the capsule and the septa. The enhancement was of low intensity. The cystic areas are not enhanced. The blood vessels originate from the diaphragm (**c**). Computed tomography (CT) demonstrated a well-defined cystic lesion of the pleura in the region of the diaphragm in the right thoracic hemisphere (**d**). The surgical findings showed a space-occupying lesion growing out of the diaphragm towards the lung (**e**). The histological diagnosis was a solitary fibrous diaphragmatic tumor without signs of malignancy. There was no recurrence in the follow-up. X-ray and CT images are courtesy of Prof. Lenz, Radiology Department Klinikum am Steinenberg Reutlingen. Surgical specimen courtesy Prof. Zimmermann, Clinic for Visceral Surgery, Klinikum am Steinenberg Reutlingen.

SFTP is a smoothly demarcated solid tumor [78,79]. On US, SFT is described as exemplary of nodular shape, smooth surface, and hypoechoic. The lung usually retains mobility in relation to the lesion [17]. Larger tumors can degenerate cystically and also show necroses. There are no data on the enhancement pattern of benign SFT on CEUS. Badea et al. described the CEUS appearance of a large pleural SFT that was found to be malignant in postoperative histology [80]. The tumor showed intense arterial enhancement beginning after 18 s. The enhancement pattern was inhomogeneous due to necroses. An intense washout began after 43 s [80]. On CT, SFT appears as a soft, hypodense mass with homogeneous contrast enhancement [54,78]. Histological confirmation by transthoracic biopsy is limited. Cordillo et al. were only able to confirm 39% preoperatively by transthoracic needle biopsy [81] [Figure 12 and Figure 13].

### 6.3. Schwannoma

Schwannomas (synonym: neurinoma or neurilemmomas) are neurogenic tumors. They are usually benign and incidental findings [82]. They originate from Schwann cells, which surround each axon and form the myelin sheath for the myelinated nerve fibers. As well as neurofibromas and perineurinomas, they belong to the benign peripheral sheath tumors (BPNST) [83]. Primary pleural schwannomas are rare and are reported to account for 1% to 2% of all thoracic tumors [84,85]. In a study of 75 cases of intrathoracic PNSTs by Boland et al, 21 cases corresponded to benign pleuropulmonary PNSTs, of which 60% were schwannomas [86]. Intrathoracically, 75% of schwannomas were localized in the posterior mediastinum, and pleural manifestation is rare [86].

Pleural schwannomas are very rarely malignant. The risk is increased with von Recklinghausen’s disease type 1 [85]. Microscopically, two different patterns are differentiated in schwannomas, which are referred to as type Antoni A and B [87]. Type Antoni A is solid and characterized by areas of spindle cell hypercellularity. Type Antoni B correlates with hypocellular areas and fluid. Histologically, schwannomas consist of spindle cells and are S-100-positive.

Hu et al. describe 11 patients with pleural schwannomas in a study. Most of them were asymptomatic. It affected patients aged 21–60 years, slightly more frequently males [82].

Schwannomas have a well-defined edge and an ovoid or round shape [82]. The mean size of the tumors was 4.4 cm (range, 2.3–6.4 cm) [86].

Schwannomas have smooth borders on US. The B-mode image can be variable depending on solid and cystic parts. The lesions may be homogeneous or inhomogeneous. The smallest vessel pixels are described in the power Doppler [88]. Larger schwannomas, in particular, if they are cystically degenerated, may have non- or hypoechoic liquid cystic parts with intraluminal septa [89].

We did not succeed in detecting CEUS descriptions of pleural schwannomas. However, there are descriptions of schwannomas in other localizations.

**Figure 14:**
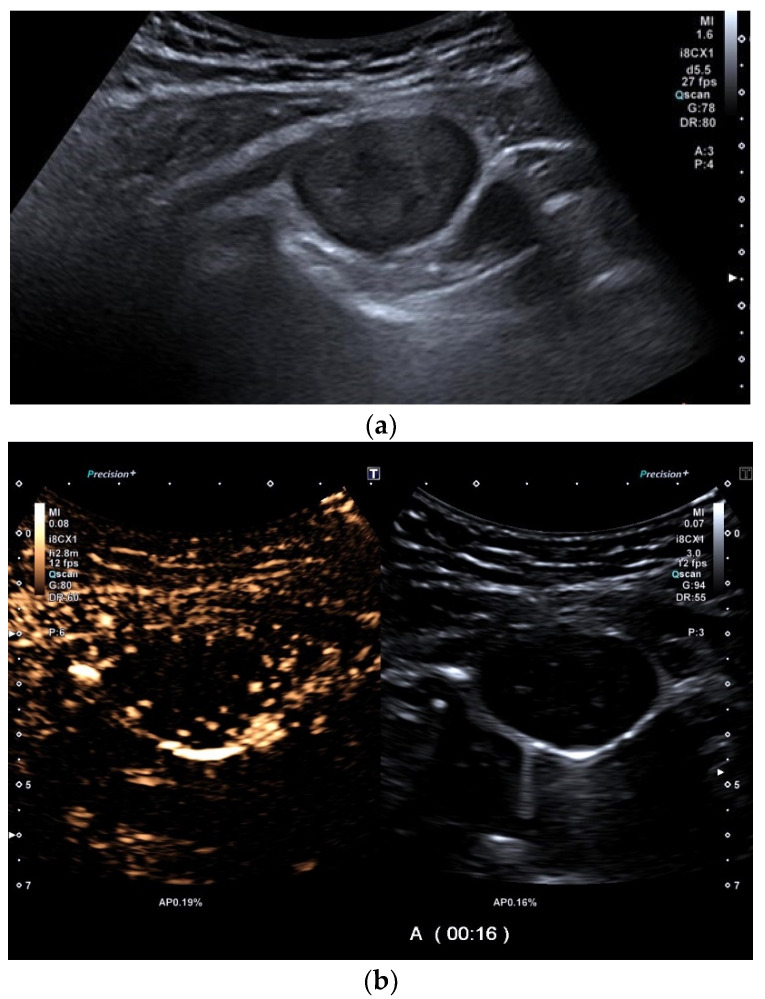

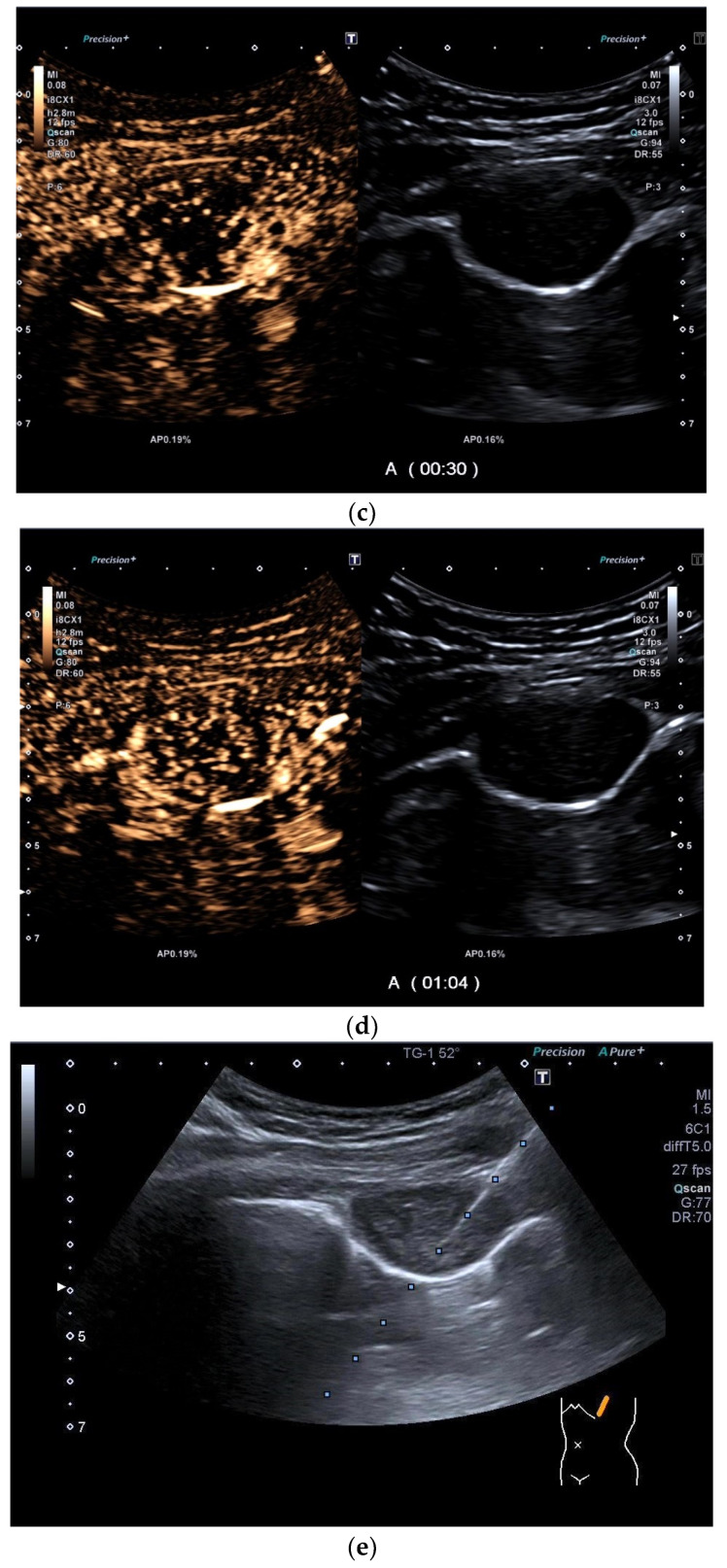
Schwannoma. A male patient with a history of smoking (20 packyears) was hospitalized for diagnostic confirmation of an unclear asymptomatic pleural lesion that has been described in a CT scan. Ultrasound revealed a small, smooth, roundish tumor at the chest wall/parietal pleura (**a**) with normal lung sliding and no signs of infiltrative growth. CEUS showed good and relatively homogeneous arterial contrast enhancement with a small central notch. The time after application of the contrast medium is indicated at the bottom of the image: A (0:16 s/ 0:30 s/ 1:04 min) (**b**–**d**). Histology from a US-guided needle biopsy confirmed a schwannoma. The illustration in B-mode-US shows the US-guided biopsy with corresponding digital planning (blue dots) (**e**).

**Figure 15:**
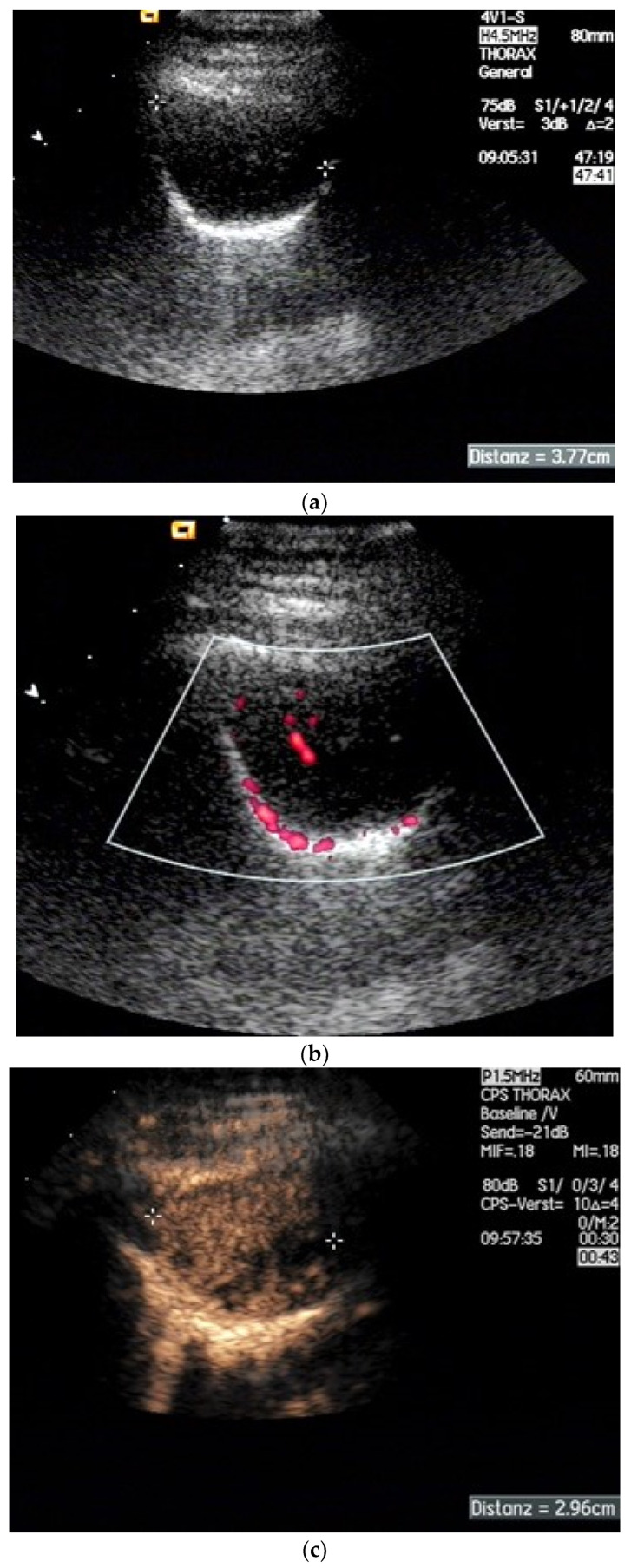
Schwannoma. As an “incidental finding”, a mass (diameter 3 cm) was found in the chest X-ray in the region of the thoracic wall, which corresponded to a solid formation on CT. The B-mode US shows a homogeneous, well-defined mass below the scapula that is positioned on the parietal pleural line (**a**). The normal lung slides over the mass. On dynamic US, lung sliding was seen on B-mode US and in color Doppler Imaging. Some vessels inside the tumor are visible (**b**). On CEUS, the lesion demonstrated mostly homogeneous enhancement in the late bronchial arterial phase. Some small areas were non-enhanced (**c**). The vascularization originated from the intercostal arteries. The lesion showed a washout only very late. US-guided sampling with a BioPince needle (1.2 mm) (Argon Medical devices company, Plano, TX, USA) was performed. Histology: Schwannoma, no signs of malignancy. Distanz—diameter.

An extramedullary dorsal schwannoma was slow and homogenously enhanced with a late washout on CEUS [90]. Schwannomas showed a moderate contrast intensity in DCE-US, less pronounced than in malignant mesenchymal tumors [75] [Figure 14 and Figure 15].

On native CT, they have an iso- or hypo-attenuated density compared to the muscles of the chest wall, with attenuations between water and soft tissue on unenhanced imaging [82,83]. In CE-CT, they show minimal or heterogeneous enhancement [82,83]. There were no specific characteristics for schwannomas on CT [82].

On MRI, schwannomas are hyperintense or isointense on T1-attenuated images and heterogeneously hyperintense on T2-attenuated images according to the regressive changes [83]. Schwannomas can degenerate cystically. Large cystic pleural schwannomas have been described casuistically [91].

### 6.4. Solitary Plasmacytoma

In contrast to multiple myeloma, solitary plasmacytoma (SP) is characterized by a single mass of clonal plasma cells without plasmacytosis and without other symptoms that are not attributable to the primary lesion. It can present either as an extramedullary (extraosseous) plasmacytoma (EMP), i.e., in soft tissue or as a solitary bone plasmacytoma (SBP) [92,93].

EMP is a rare disease but can occur in any organ. Approximately 30% of patients with EMP develop MM within 10 years of initial diagnosis [92].

The solitary extramedullary plasmacytoma consists of a soft tissue mass that is not in contact with the bone [93]. Minimal infiltration of the bone marrow by clonal plasma cells (PC < 10%) was nevertheless considered consistent with an SP diagnosis [92].

The diagnosis of SP is based on histologic and immunohistochemical evidence of homogeneous infiltration of monoclonal plasma cells with CD138 and/or CD38 expansion.

MRI can detect soft tissue and bone marrow lesions and is the gold standard for detecting spinal cord compression. CT can be helpful for locoregional staging. On MRI, the solitary plasmacytoma appears as an infiltration with a low T1 and a high T2 signal intensity.

**Figure 16:**
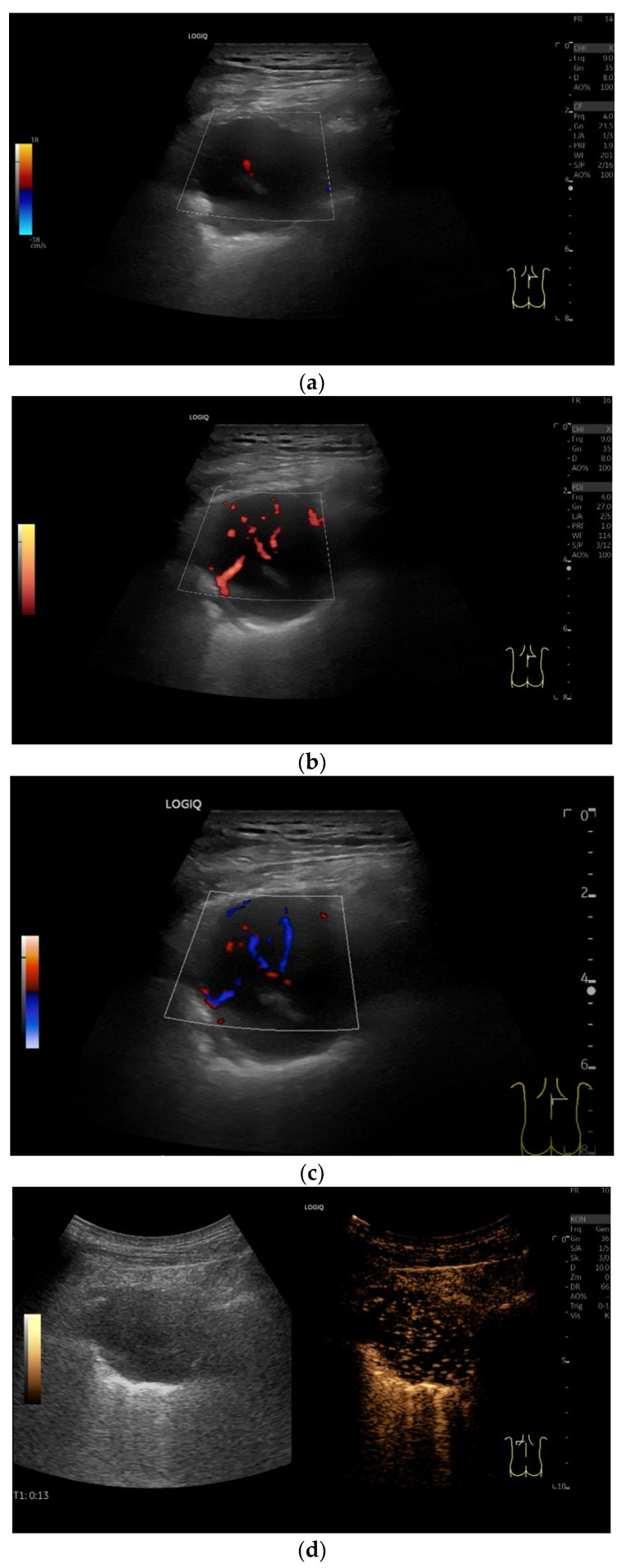

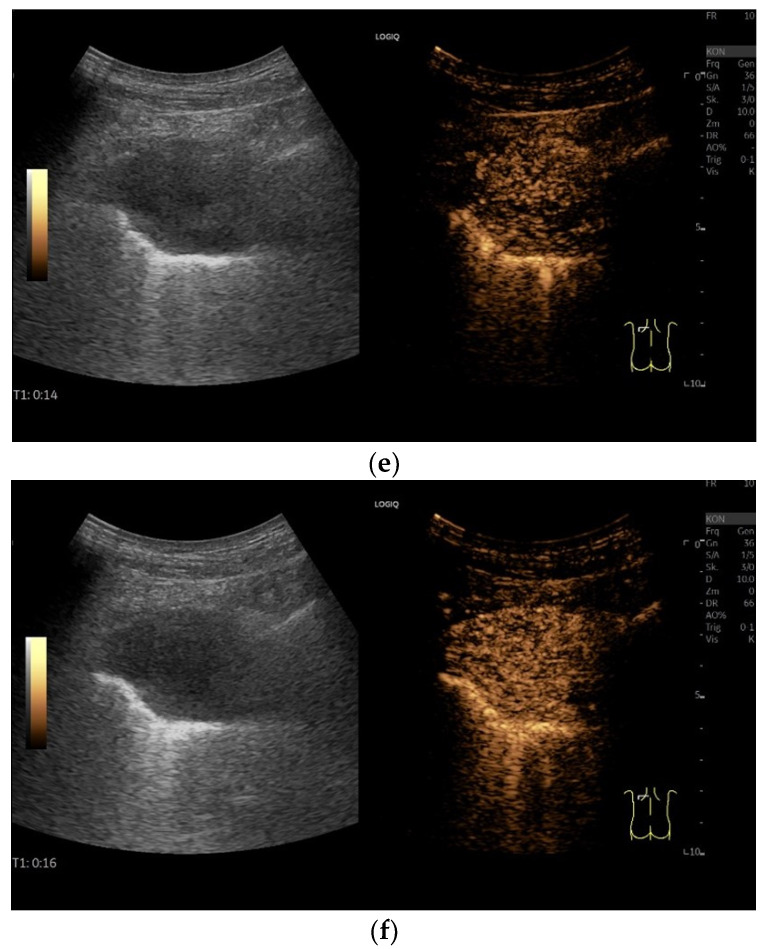
Solitary extramedullary plasmacytoma histologically confirmed. Male patient with weight loss. Chest CT revealed a right paravertebral mass in relation to the chest wall, pleura, and lung. Ultrasonography showed a highly hypoechoic, almost non-echoic lesion. This displaces the lung. An echogenic reflex is seen centrally, and a vascular structure is seen in the color Doppler Imaging (**a**). Multiple macrovessels are delineated in the power Doppler and bidirectional power Doppler (**b**,**c**). On CEUS, the first signals arrive at 13 s in the bronchial arterial phase (**d**). The lesion is homogeneously enhanced in the bronchial arterial phase (**d**–**f**). Histologic confirmation was performed by percutaneous US-guided needle biopsy. T1- time after application of ultrasound contrast agent.

Ding et al. describe the appearance of extramedullary plasmacytoma on CT and MRI in 10 patients in different organs [94]. Only two of the masses were located in the posterior thoracic wall. The masses appeared very different: The tumor shapes were both rounds, stripped, irregular, and nodular. The margin of the tumors was well-defined or only partially well-defined. Cystic degeneration was seen in 2/10, and calcifications in 1/10. The CT attenuation and MR signal intensity of tumors were of similar proportions, heterogeneous and homogenous. With contrast injection, a marked enhancement was usually seen, more rarely a mild, mild to moderate, and delayed enhancement [94]. Findeisen et al. demonstrate CEUS of a solitary plasmacytoma with homogeneous arterial enhancement at 31 s [12]. We have not been able to find further descriptions of extramedullary solitary plasmacytoma in CEUS [Figure 16]. With regard to the special complex radiological and hematological diagnostics as well as the therapy, we refer to the guidelines of the European Expert Panel [92].

Key features of different benign pleural lesions on TUS are summarized in [Table 2].

## 7. Conclusions

TUS is a real-time performance to investigate the pleura, detect pleural thickening, pleural effusions, pleural tumors, and peripheral lung lesions, and describe them in B-mode US, CDI, and CEUS. The advantages of the procedure are the dynamic character of the examination, the high resolution, and the combination of various ultrasound techniques. The pleura can be assessed on TUS because it is adjacent to the chest wall and is not obscured by lung artifacts. Exceptions are the mediastinal pleura and the pleural parts located in the dorsal acoustic cancellation behind the ribs. Even though CT is the standard procedure for diagnosing pleural and pulmonary diseases and tumors, ultrasound offers valuable information and can be used in the initial diagnosis, co-assessment, or follow-up of pleural thickenings, pleural lesions, and pleural and peripheral pulmonal tumors. TUS can be used to determine the thickness of the pleura, identify internal structures in effusions, determine pleural masses, and draw conclusions about the cause. By observing the pleural sliding, a distinction can be made between pleural lesions and peripheral lung lesions.

Benign pleural thickenings are usually thinner than 10 mm and have a uniform appearance. Malignant pleural thickenings are often more pronounced, thicker than 10 mm, and usually irregular, wavy, or nodular. Inflammatory/infectious diseases such as empyema and tuberculous pleurisy, but also post-traumatic hemothorax and, in the course of the disease, a residual fibrothorax show pleural thickening to varying degrees and fibrin strands.

In systemic diseases such as IgG4-associated disease, sarcoidosis, amyloidosis, and connective tissue diseases, pleural involvement is rare, and ultrasound descriptions are hardly available. In the clinical context, however, the possibility of using TUS in the case of pleural effusion or corresponding clinical questions should be considered.

Pleural plaques are oval, hypoechoic, homogeneous lesions in US. Thoracic splenosis should be considered after accidents involving splenic trauma, splenic rupture, or splenectomy. These are round, homogeneous masses that are usually very easy to diagnose on CEUS in other locations. A typical characteristic is the long-lasting contrast enhancement typical of the spleen, lasting several minutes. A catamenial pneumothorax or hemothorax is suspected of thoracic endometriosis. There are no descriptions on TUS or CEUS. The merit of TUS is to diagnose a catamenial hemothorax in the overall context. An attempt can be made to detect the small endometriosis nodules on TUS. However, the diagnosis, in combination with therapy, is made thoracoscopically. Mesothelial cysts present with typical cyst criteria. If the content is not clearly anechoic in cases of doubt, non-enhancement can be detected using CEUS.

There are not many descriptions of the appearance of benign pleural tumors on TUS and CEUS. Thus, we are cautious about generalizing the descriptions. However, lipomas are described as hypoechoic with only slight and heterogeneous enhancement on CEUS. Benign SFT appears hypoechoic, may contain cystic areas, and is well vascularized and hyper-enhanced on CEUS. Differentiation from malignant SFT is not possible on the basis of TUS and CEUS. Schwannomas are homogeneous, hypoechoic, and may have cystic parts. On CEUS, the solid parts are hyper-enhanced. The solitary extramedullary plasmacytoma is hypoechoic on TUS and hyper-enhanced on CEUS.

The importance of CEUS in pleural masses lies in the differentiation of nonenhanced liquid encapsulated effusions, cysts, and necroses from solid tissue and in supporting US-guided biopsy. Malignant pleural thickenings often show faster and more intense enhancement in CEUS. However, CEUS alone cannot differentiate between benign and malignant pleural thickenings. In the overall context, however, CEUS offers valuable additional information.

In the case of pleural masses, a decision must be made as to whether a US-guided biopsy should be performed or whether surgical resection is primarily indicated. Surgical therapy is not indicated for every tumor, but systemic therapy for secondary metastases or radiotherapy for solitary myeloma, for example. If a malignant pleural tumor with necrosis is initially suspected, it may be useful to perform US-guided sampling under CEUS.

We advocate the inclusion of TUS and CEUS in the diagnosis of pleural thickening, lesions, and tumors whenever possible and clinically appropriate.

## Figures and Tables

**Table 1:** Pleural lesions, masses, and thickening.

Benign	Malignant
***Infectious/inflammatory, post-traumatic:*** Empyema tuberculous pleuritis hemothorax fibrothorax ***Systemic diseases:*** IgG4 related diseases Sarcoidosis Amyloidosis Connective tissue diseases, vasculitis	Metastases Malignant pleural mesothelioma Malignant SFT Sarcoma
***Tumor-like lesions:*** Plaques Diffuse pleural thickening associated with asbestos exposure Splenosis Endometriosis Mesothelial cysts Lymphangiomatosis	
***Benign tumors***: Lipoma Benign SFT Schwannoma Solitary extramedullary (extraosseous) Plasmacytoma	

**Table 2:** Key features of different benign pleural lesions as visualized by TUS.

Pleural Lesion	Appearance in TUS (and CEUS)
Benign pleural thickening	Usually less than 10 mm thick, evenly thickened, no nodular thickening
Empyema	Hypoechoic thickened pleura, internal echos in the pleural effusion, hypoechoic pus, hyperechoic gas reflexes, fibrin strands, and chambering. In the phase of organization, thickened pleura, chambering of the pleural space.
Empyema necessitans	Exceeds the parietal pleura and infiltrates the surrounding soft tissue and chest wall muscles.
Tuberculous pleuritis	Pleura effusion with fibrin strands, pleural calcification, and thickening. Hypoechoic granulomatous inflammation and granulomas with hyper-enhancement on CEUS in thickened pleura. Hypoechoic caseous abscesses in thickened pleura, hypo- or nonenhanced, heterogeneously enhanced lesions, with contrast-enhanced septations and contrast-enhanced rim.
Hemothorax	Pleural effusion, blood appears as hypoechoic content. Pleural thickening, fibrin, and septa may form.
Fibrothorax	Extensive and dense fibrosis of the visceral pleura, with fusion of the visceral and parietal pleural layers, no lung sliding.
Encapsulated pleural effusions	Round, smoothly circumscribed hypoechoic or anechoic masses. No evidence of macrovessels in CDI. Non-enhanced in CEUS.
Plaques	Ovoid, hypoechoic, homogeneous lesions. Calcifications are possible.
Splenosis	Round, homogeneous lesions. Macrovessels on CDI are possible. Spleen-typical contrast behavior in CEUS with long-lasting contrast enhancement over several minutes.
Thoracal endometriosis	(Catamenial) hemothorax (and pneumothorax).
Mesothelial cysts	Typical cyst criteria. If the content is hypoechoic and not anechoic due to near-field artifacts, CEUS is helpful. Non-enhanced in CEUS.
Lipoma	Homogeneous or heterogeneous hypoechoic mass without any calcification or internal vascular supply. Slight heterogenous hypoenhancement on CEUS.
Benign solitary fibrous tumor	Smoothly bordered, hypoechoic. Examples with a nodular shape have been described. Larger tumors can have cystic parts. With few data and based on our example, the solid parts are hyper-enhanced in CEUS.
Schwannoma	Round or oval, smoothly bordered, hypoechoic. Cystic parts are typical, especially in larger tumors. In CEUS, the solid parts are hyper-enhanced.
Solitary extramedullary plasmacytoma	Round, hypoechoic tumor. Macrovessels on CDI. Homogeneous hyper-enhancement on CEUS.

## Data Availability

No new data were created or analyzed in this study.

## Supplementary Materials

The following supporting information can be downloaded at https://www.mdpi.com/article/10.3390/diagnostics15020176/s1, Video S1. A video loop shows the hypoechoic tumor in the CDI with macrovessels and during quiet breathing with the lung sliding over the pleural tumor.
